# Experiences of nursing and medical students with the use of Artificial Intelligence tools such as ChatGPT in Uganda

**DOI:** 10.1371/journal.pgph.0005715

**Published:** 2025-12-30

**Authors:** Ritah Nantale, Frank Kayemba, Kennedy Paungholi, Joshua Epuitai, Brian Jemba, Elizabeth Ajalo, Adam Afodun, Milton W. Musaba, Ian G. Munabi, Sarah Kiguli, Aloysius Gonzaga Mubuuke, David Mukunya

**Affiliations:** 1 Faculty of Health Sciences, Busitema University, Mbale, Uganda; 2 College of Health Sciences, Makerere University, Kampala, Uganda; PLOS: Public Library of Science, UNITED STATES OF AMERICA

## Abstract

Medical and nursing students widely use ChatGPT and other artificial intelligence (AI) for their educational and learning needs. However, there are concerns of misuse and abuse of AI tools in medical education. We explored the experiences of medical students with the use of ChatGPT and other AI tools in education. This was a qualitative descriptive study to explore experiences of medical students, including students of Bachelor of Medicine and Surgery, Bachelor of Science in Nursing, and Bachelor of Science in Anaethesia at Busitema University in Uganda. We conducted five focus group discussions comprising 8–10 participants and 7 in-depth interviews. Participants were purposively selected across the three programs and were spread across the different years of study. Data were analyzed using thematic analysis with the aid of Atlas ti.9 software. Four themes were identified: 1) experiences of students with the use of AI; 2) perceived benefits of AI tools; 3) concerns regarding AI use; and 4) recommendations for responsible AI use. Students noted experiences with using AI as an initial source of information, while some used it as a last resort or concurrently with other traditional sources of information. AI was used for various learning needs, including preparing for tutorials and examinations, writing research proposals, and answering examination questions. Perceived benefits included enhanced learning, readily access to accurate and simplified information, saves time, provides immediate feedback, and improving scholarly writing and preparation for examinations. Students’ concerns with AI use included questionable accuracy, credibility, unreliability, inherent AI limitations, and problems arising from over-reliance on AI. Modifying examinations and assessments, changing attitude towards AI use, and using other resources alongside AI were recommended to result in responsible AI use. Participants had positive perceptions about the use of AI but also expressed fears regarding its use in medical education. Structured curricula, formal policies, and guidelines are needed to adequately prepare medical students for the integration of AI in medical education.

## Background

Internationally accessible end-user Artificial intelligence (AI) applications have impacted medical education worldwide [1]. Generative AI chatbots, such as ChatGPT and Gemini, are among the most used applications. These natural language processing models generate human-like texts and have the potential to empower medical education [2,3]. They can serve as virtual teaching assistants, providing students with alternative means of accessing information, and can be used in academic writing, patient care, and aid in decision-making making [4–7]. AI-powered chatbots can also provide medical students with personalized learning experiences, tailored to their learning needs and supported with immediate feedback [8]. This can enhance learning outcomes and ultimately improve patient care [8].

The benefits of AI do not come without challenges. There are several ethical and legal concerns regarding AI use, such as privacy and security of data, copyright infringements, bias, plagiarism, and incorrect responses or fabricated data from AI tools [6,9–11]. Additionally, AI could also undermine the academic merit if used for examination malpractice [12]. As such, AI policies and guidelines are needed to ensure the responsible use of AI tools among students [13]. Constructing these policies and or guidelines requires knowledge about students’ use, experience, and perception of AI in medical education.

The majority of nursing and medical students are using AI in their education [14,15]. However, its perceived effects on health professions education have not been explored, especially in low and middle-income countries (LMICs) [16]. This study, based in Uganda, bridges the gap by exploring the experiences of nursing, anesthesia, and medical students with the use of AI tools such as ChatGPT in education. The study was focused on exploring experiences, perceived benefits, challenges students faced with the use of AI in nursing and medical education and the proposed recommendations to ensure responsible AI use in medical education.

## Methods

### Study design and setting

This was a qualitative descriptive study that explored the experiences of undergraduate nursing and medical students with AI use [17]. The study was conducted at Busitema University Faculty of Health Sciences, a public University in Mbale city, eastern Uganda. The faculty was established in 2013 and offers undergraduate training to about 500 students in three programs: Bachelor of Medicine and Bachelor of Surgery, Bachelor of Science in Nursing, and Bachelor of Science in Anesthesia.

### Study population and sampling

The study participants were undergraduate nursing, anesthesia, and medical students. These included students who were studying Bachelor of Science in Nursing, Bachelor of Science in Anaesthesia, and Bachelor of Medicine and Surgery at Busitema University. Students were purposively selected across the three programs and they were spread across the different years of study. The variation of the participants was based on demographic composition, such as age, gender, and course of study. The sample size was based on the principle of saturation [18].

### Data collection

Data were collected between 06^th^ November 2023 and 31^st^ January 2024. We conducted five focus group discussions (FGDs) (one per year of study), including 8–10 participants and seven in-depth interviews. We used an interview guide designed based on Joan Hughes RATs model to explore students’ experiences with the use of AI (Appendix 1). Interviews were conducted face-to-face, in a private room to avoid distractions and facilitate clear recordings. Data were collected by authors R.N, and D.M, who have experience in qualitative data collection and were fluent in the English language in which interviews were conducted. We explained the study’s purpose to the participants, obtained their consent, and collected demographic information before starting any discussion or interview. We assigned participants random numbers as codes before collecting data for anonymity. The interviews and discussions lasted for about 30–60 minutes. Detailed hand-written notes were taken by the note-takers, and the interviews/discussions were audio-recorded with permission from participants.

### Data analysis

Data was analyzed using inductive thematic analysis following the six steps proposed by Braun and Clarke (9). The first step was to read the transcripts thoroughly to become familiar with the data. The second step was to identify meaningful statements from phrases and sentences to generate the initial codes. During the third step, we identified the potential themes and sub-themes by combining the appropriate codes into categories. The fourth step was to review and modify themes by scrutinizing and comparing the codes within a given theme as well as with codes in other themes. We then defined and renamed the themes as necessary. The final step was to prepare the report by selecting obvious examples and reporting the quotes in the participants’ own words without any change to ensure precision. We used ATLAS.ti version 9.0 to organize the data. Data were analyzed by authors R.N, K.P, and F.K.

### Ethical consideration

The study was conducted according to the Declaration of Helsinki and in line with the principles of Good Clinical Practice and Human Subject Protection (10). Ethics approval to conduct the study was granted by the Busitema University Research Ethics Committee (BUFHS-2023–79). Written informed consent was obtained from each student before recruitment into the study. There was no coercion to participate in the study, and participants were free to withdraw at any time. Participants were anonymized by assigning them unique codes as identifiers. Interviews were conducted in a private research office setting outside the University premises. The data collected was kept in a locked folder on the computer’s hard drive, which was password-protected and only accessible to the investigators.

## Results

### Description of the participants

A total of 50 students participated in the interviews. The median (interquartile range) age was 22.8 (2.3) years (Table 1). Most (56.0%; n = 28) were males, and 70% (n = 35) were Bachelor of Medicine and Surgery students.

**Table 1:** Description of the participants.

Variable	Frequency (%)
**Age in years (median and interquartile range)**	22.8 (2.3)
**Sex**	
Male	28 (56.0)
Female	22 (44.0)
**Program**	
Bachelor of Science in Nursing	9 (18.0)
Bachelor of Anaesthesia	6 (12.0)
Bachelor of Medicine and Surgery	35 (70.0)
**Year of study**	
First year	9 (18.0)
Second year	10 (20.0)
Third year	11 (22.0)
Fourth year	10 (20.0)
Fifth year	10 (20.0)

### Themes

Four themes were identified from the study: experiences of medical students with generative AI, perceived benefits of AI, concerns about its use, and recommendations for responsible AI use (Table 2).

**Table 2:** Describes the themes and subthemes of the study results.

Theme	Subtheme
Experience of medical students with generative AI	Use of AI tools among nursing and medical students
	Ways in which AI tools were used
	Uses of AI among students
Perceived benefits of AI	Facilitates learning
	Simplified, accurate, and updated information
	Examination preparation
	Scholarly writing
	Gives students immediate feedback
Concerns about AI use	Questionable accuracy
	AI is user-dependent
	AI tool limitations
	Fears of overreliance on AI
Recommendations for responsible AI use	Use AI alongside other resources
	Attitudinal change to embrace AI
	Modify types of assessments

### Theme: Experiences of medical students with the use of AI tools

#### Subtheme: Use of AI tools among nursing and medical students.

During interviews, students reported wide use of AI tools. Commonly used AI included ChatGPT, Bard (Gemini), Write Sonic, Quill Bot, Julie Pot, and Robot, and virtual assistants such as Google Assistant, Alexa, and Siri. Students were introduced to ChatGPT and other AI tools by their classmates, friends, tutors, lecturers, while some were introduced through social media or other media platforms. Students also recommended AI tools to people within their circles.

*“… for ChatGPT, it was a year two friend who recommended it to me; he sent me a link… now for Bard, I think I was doing some work, and literally ChatGPT wasn’t giving me the reliable information I wanted. Still, I have a friend who is in his fourth year. He told me; man, just try out Bard, it is nice.” (21-year-old male first-year medical student R1 FGD 3)*

*“I got to know about Chat GPT in a lecture. So, our lecturer came, he was teaching ICT. He did it practically. I installed the app on my phone.” (25-year-old female first-year medical student R2, FGD 3)*

#### Subtheme: Ways in which AI tools were used.

Some students used AI as their first source of information for their reading. AI was preferred as the initial source of information as it enabled them to understand unfamiliar subjects in a short time. However, some students used AI at the last minute or as a last resort, when they had a short time left before an examination or to submit their assignment. In this context, AI was seen to provide a quick summary of the subject content and direct answers to the students. AI was also used concurrently with other reliable sources of information. AI would give students an overview of the subject matter, and students would use textbooks for detailed and comprehensive information.

*“I don’t waste much time on hard concepts. If something is hard for me, I just go to the AI and I find out the information much quicker. Unlike the times back when something was hard in the textbook, you go to Google, and they give you just related information, not the exact information you need.” (24-year-old fourth year medical student, IDI, 103)*

*“These AIs are actually... something great. They’ve made some of our lives easier, so when you’re in a dilemma, like you’re faced with a lot of work to do, and you’re not sure whether you will manage to go and open textbooks. You can easily get some clue, you just open your AI, your ChatGPT, and you get a clue.” (22-year-old male second-year medical student, R4, FGD 5)*

While the majority of students used AI for searching health-related information, some used it to search for non-medical-related information. Others used it for leisure, emotional support, and stress management. During the COVID-19 pandemic, when students were in social isolation, AI was used for therapeutic conversation, chatting, and music therapy. The conversational nature of AI tools enabled students to connect with them emotionally and share intimate information.

*“… but during that time, I was not using it for medical stuff, but outside medicine. Computer stuff. Generally, computer programming and computer science.” (21-year-old male first year medical student R7, FGD 3)*

*“…. Let’s just say, no one wants to talk to you, but you have artificial intelligence, it really helps because for me it plays me music. It’s like interacting with another person; you can even converse with it. You tell it like this. It will tell you this. Like it will respond in a way and you really feel so good” (22-year-old male second year medical student R6, FGD 5)*

#### Subtheme: Uses of AI among students.

Students primarily used AI for reading and for completing their assignments. While students traditionally used lecture materials, textbooks, and online sources for their reading, they have partly replaced traditional sources of information with AI. Students preferred AI for its ability to provide direct answers, ease of use, specificity, and accessibility. Searching for information through traditional sources was thought to be a cumbersome and tedious process, yet it was not the case with AI. Students used AI for last-minute reading and revision for examinations. Students reported that some of the multiple-choice questions from the AI appeared in the examinations, which further cemented their preference for AI for examination preparation. Students also reported using AI for writing their research proposals, grants, and projects. AI was noted to provide all the relevant write-ups for the students, including references for citing.

*“So, instead of going to the textbook, A textbook gives a lot of work, it’s bulky. But, if you go to ChatGPT, it gives you direct answers to the objective that you want…” (22-year-old male second year medical student R1, FGD 5)*

### Theme: Perceived benefits of AI sources

#### Subtheme: Facilitates learning.

Students reported that AI has made learning easier and quicker. They use it to expound on what they have learned and understand complex terms from textbooks. Generative AI was used as a personal tutor to clarify concepts that were not well understood during lectures. Learners who were timid to ask questions in class opted for ChatGPT. Students use AI when they want to teach themselves complicated concepts and when they fail to find information elsewhere. Students also noted that ChatGPT makes learning interactive because it gives the user feedback, unlike a textbook.

*“It has actually eased studying because… I rarely ask questions in class, but I always have questions in my head... I would just go and type it in ChatGPT and then look through the responses. (22-year-old male second-year medical student, R4, FGD 5)*

*“The reason why some people use it is because they’ve failed to find information elsewhere. Someone has tried textbooks and trusted sources, and failed. Then they try ChatGPT, and it gives them the response.” (22-year-old second year medical student, R6, FGD 5)*

Students can use AI to augment their clinical skills by getting information about clinical procedures.

*“…you don’t know how to do gastric lavage because you’ve never handled a poisoning case, you just type it in AI. It brings the procedure immediately, which is helpful in emergencies.” (25-year-old female first-year medical student R2, FGD 3)*

#### Subtheme: Simplified, accurate, and updated information.

Health professions students found AI tools user-friendly. AI presented information in a simple format that was easy to understand, more goal-oriented, and more precise. Students used AI as it was easier to comprehend information in AI than from textbooks.

*“You see, some textbooks have complicated language, you read something and you fail to understand easily... AIs normally have simplified language. So, I go there... Maybe I can get some clue, then go back and revisit the book and understand.” (22-year-old male second year Anaesthesia student, R5 FGD 5)*

#### Subtheme: Examination preparation.

Besides facilitating learning, AI was seen to help students prepare for examinations, tutorial presentations, and completing course assignments. Some used AI for answering continuous tests given online. AI would give them answers to specific questions instantly and also generate MCQs to pretest themselves before the exams.

*“The way you prepare for an exam changes, most especially at the last hour. At the last hour, for me, I mostly use this AI for WhatsApp, I just type there what I want to read last, and then it gives me direct answers” (21-year-old male second year medical student, R3, FGD 5)*

*“I wasn’t prepared. I think out of the 50 questions, I was so confident of around 20 … the 30 questions which I wasn’t confident about, it helped me generate answers and I passed well.” (24-year-old male fourth year medical student IDI, 103)*

AI sources were seen to be more efficient for meeting deadlines and submitting assignments on time. Students found AI to be convenient and applicable in “emergency” situations. For example, when they needed to read or revise in a short period for exams and tutorials.

*“It helps beat deadlines, because if you don’t have time, it will save you from reading a lot, because if you read a lot, you need to filter your work and all that.” (25-year-old female first-year medical student, R2, FGD 3)*

*“I feel it is helping because usually our schedules in medical school are so busy, like you have one day to prepare for exams, and there are many things. So, if I’m going to revise that textbook for an exam, it may be hard. But if I have an AI just asking your particular questions. I think it’s easing the work in one way or the other” (22-year-old male second year Anaesthesia student, R5, FGD 5)*

#### Subtheme: Scholarly writing.

Students utilized AI in research to write research proposals, grant applications, and official documents such as resumes. ChatGPT made writing research proposals easier for students, as it would write the entire proposal. Some were actually using it to write grant applications. Additionally, some were using it to understand some concepts in research, such as data analysis.

*“We typed in our research question, instructed ChatGPT to help us generate a research proposal, which it did. It wasn’t so much detailed; however, it was able to give us the highlights and the way the proposal is supposed to flow.” (24-year-old male fourth-year medical student, IDI, 103)*

*“It has just guided me on how to, for example, apply for grants... But for grant application it has.” (22-year-old male second year medical student R6, FGD 5)*

Students preferred Bard and Write Sonic when they needed references for further reading or making citations. AI sources provided reliable references, which were often good and saved browsing time for students.

*“That is how I ended up using Write Sonic, and it is doing me very well, yes, with references,* it was a green flag for me.” *(21-year-old female first-year nursing student, R5, FGD 3)*

#### Subtheme: Gives students immediate feedback.

Students preferred ChatGPT because it provided them with immediate feedback and tailored responses to their questions. Students also found ChatGPT to be an interactive tool with which they can have a conversation.

*“I have also used ChatGPT a couple of times, and my experience is that whenever you start somewhere, it feels like you’re interacting with someone. You feed in a question, and it brings the answers immediately.” (24-year-old female fifth year medical student, R6, FGD 1)*

### Theme: Concerns about AI use among students

#### Subtheme: Questionable credibility of AI sources.

Although students believed that the information from AI was accurate, most of them thought that AI sources were not credible sources of information. Students were not confident in citing AI sources as their reference materials. Students also perceived that some of their lecturers were against and openly discouraged the primary use of AI. Students thought using AI was similar to relying on Wikipedia, which would not result in a competent healthcare provider.

*“I’m quite sure if they give us the assignment, and we are required to indicate the references, I’m sure no one will write ChatGPT as a reference.” (22-year-old male second-year medical student, R4, FGD5)*

*“Of course, lecturers now have a great bias about AI… they’ve actually outlined for us the reliable sources where we’re supposed to get the information, the articles we are supposed to read, and the various sources, where we should get information that is trusted. So, when you come… I’m sure when you are in a tutorial, and then you present your research, and then you give your reference as ChatGPT. I’m sure it will not make sense to the lecturer. (22-year-old male second-year medical student, R4, FGD 5)*

#### Subtheme: Questionable accuracy of AI sources.

Generative AIs were reported to be unreliable as they sometimes provided wrong and inaccurate information. Occasionally, AI sources provided generic responses with unnecessary and technically irrelevant details. Students reported that AI sources lacked precision and technical details that they believed were critical for a field like medicine/nursing, where human life was at stake. The information from AIs lacked local context; therefore, they could not adapt their response to the local setting. Some students also noted that information from AI sources may be outdated. AI tools may not keep pace with the current information.

*“Then another fear is the accuracy of the information it gives….in the medical field…we need great precision of the information in that we are dealing with life. The information has to be as accurate as needed. So, if it gives you wrong information…, you may find it is affecting you a lot” (24-year-old male fourth year medical student, IDI, 103)*

*“For example, some data – like the epidemiology of obstructed labour in Eastern Uganda – is not going to come. It will tell you, I’m not sure about that, but maybe data for America. It’s because our countries are behind in keeping data, in putting information online, and they definitely deprive us of getting the information.” (24-year-old male fifth year medical student, R2, FGD 1)*

Students reported experiencing negative consequences of getting inaccurate information from AI sources.

*“I tried to use it in research, and I failed terribly because it was giving me things that were not coming up very well…I also used it as an alternative to the textbooks, which also didn’t work as well.” (22-year-old female second-year medical student, IDI 101)*

*“…. When you tell it to cite for you, it may cite wrongly. So, it can decide to do secondary citing for you, whereby it cites a paper that was cited by another person. So, if at all, the other person made a mistake. And you also. Use the same paper. It can end up leading you into medical legal issues.” (22-year-old male second year medical student R6, FGD 5)*

#### Subtheme: AI is user-dependent.

Students noted that while the information in AI sources was vast, its accessibility was dependent on the individual user. The information accessed depended on the specificity and precision of the prompts.

*“…but the phrasing of the question also matters because you have to phrase it in a way that you can get the actual information ……They are going to bring you a lot of information that is not very specific to your objective” (21-year-old male second year medical student, R3, FGD 5)*

#### Subtheme: AI tool limitations.

Students reported that Gen AI tools had limited capabilities. They could handle only one prompt at a time and hence could not multitask. Some AI sources failed to process the prompt. Most AIs were limited to text prompts and responses; they could not process or produce graphical data. They could not do accurate calculations either.

*“What I have noticed about ChatGPT, it cannot multitask. For example, if you send in a command for a calculation or something else, then immediately after that, before it responds, you send in a command to write an essay. It cannot do it at the same time, so it needs you to send one thing at a time.” (21-year-old female second year medical student R2, FGD 5)*

AI sources were also limited to online use. This was seen as costly for students because of the requirement for internet access. Students could not afford the premium AI or subscription-based service that has enhanced features for their learning.

*“It requires data (internet access), and not every time one will have data, so I would opt for textbooks.” (22-year-old female second year medical student*, R8, FGD 5)

#### Subtheme: Fears associated with overreliance on AI tools.

Students feared that they would be accused of examination malpractice due to AI use. They recalled scenarios where students were charged with examination malpractice due to the use of AI. Using AI was feared to breed mistrust from lecturers and reduce the credibility of online tests.

*“I think my only fear also about this thing maybe getting caught. Second years were caught… You can be caught if you’re using it, like if you’re entirely depending on it” (20-year-old female nursing student, R4, FGD 3)*

Students anticipated that free AI versions could be withdrawn with time, given that AI needs a lot of system resources, and companies will be forced to monetize them fully.

*“I think after they obtain data on its performance, the fee will be raised because there is a version that I wanted to upgrade to, they told me you pay... Will it be there for a normal student who … doesn’t have payment?” (21-year-old male first-year Anesthesia student, R3, FGD 3)*

The health professions students worried that AI would breed laziness, procrastination, and overdependency on it. Some believed that using AI reduces critical thinking and creativity, which would reduce the competence of health workers. They fear that overreliance on AI sources would result in incompetent healthcare providers.

*“So, these AIs, the way they work, I think they cut out that part of the students being creative. You can’t think because AI is there.”* (*22-year-old male second year Anesthesia Student, R7, FGD 5*)

*“I can’t take my patient to a doctor who has been using ChatGPT because I personally know that its information is insufficient. It’s good to a certain level. It’s not like complete, it’s not equivalent to what the textbooks give.” (22-year-old male second year Anesthesia Student, R7, FGD 5)*

Lastly, students were worried that AI would take over the jobs of some people. They also raised concerns about data privacy. Students thought that their personal information may be misused for various reasons.

*“One of the fears it can be good at replacing people who are professionals in certain fields. For example, if there are people who are experts in writing grants, writing project proposals…” (24-year-old male fourth year medical student, IDI 103)*

*“Another thing it asks for much information even when you are coming in to register. Can’t other people access that information like we hear of hackers?” (21-year-old male first year Anesthesia student, R3 FGD 5)*

### Theme: Recommendations for responsible AI use

#### Subtheme: Use AI alongside other resources.

Students suggested that AI should be blended with other sources of information. Students also suggested comparing the work of different AI sources to verify the validity, reliability, and accuracy of the information. We also require human oversight to proofread and edit information from AI sources, including verifying references and citations.

*“We don’t need to rely on this ChatGPT for our learning. Yes, we need to have it alongside the books, but our main source of information should be the books, and then maybe ChatGPT can come in at the time when it should come. Maybe you need something urgent to ask.” (22-year-old male second year medical student R7, FGD 5)*

*“Usually, I do the work on Chat GPT and then compare with Google Bard, and then I go for other recommended textbooks and other reliable sources like peer-reviewed work... So that’s how I try to balance the use of ChatGPT, and others like to make the information more accurate.” (21-year-old male first-year medical student, R7 FGD 3)*

#### Subtheme: Attitudinal change to embrace AI.

Students noted that the faculty were against AI for the wrong reasons. The students believed that lecturers were blind to the benefits of AI and the fact that its use was inevitable, and it’s here to stay. They thought that sensitizing and training the faculty could help change their attitudes towards AI. This was thought to enable faculty to embrace AI use in their teaching. It would also require students to be trained on how to use AI optimally.

*“So, the first point is to encourage people; to tell them that it’s OK to use ChatGPT, but it’s not enough, so you use ChatGPT and then you also use other sources to compare the information and to consolidate more. So, like that would be of a better version.” (22-year-old male second-year medical student, R4, FGD 5)*

*“These lecturers think that when they give the work, students just go and ask the AI… I think explaining to them how AI can be used as a tool to enhance productivity will make them more inclined towards accepting it.” (21-year-old male first year medical student, R7, FGD 3)*

#### Subtheme: Modify the type of assignments.

Students thought that the faculty should stop giving online tests in light of examination malpractice from AI use. They recommended that strict supervision of online supervision would be critical for tests that are required to be done online. The use of AI detectors was also suggested to detect AI misuse in research proposals and assignments. Students suggested the use of practical examinations, viva voce examinations, and application-based questions like case scenarios, where AI can’t provide answers to students.

*“… I think they can set these questions more with maybe scenarios. Yes, if they are scenarios, maybe it could be hard for the AIs to figure it out. I must think and use a lot of logic to answer this scenario case… I think they should do away with their online exams because those ones, really. How do you tell me the exam is online? Like if I’m stuck, I will literally go to ChatGPT” (20-year-old female, first-year nursing student, R4, FGD 3)*

*“I would think that when you are giving an online assessment, you would require…the use of plagiarism checkers… checking whether the answers are generated by AI would be good.”* (*22-year-old female second-year medical student,* IDI 101).

## Discussion

The study explored the experiences, of medical, nursing, and anesthesia students regarding the use of AI tools during their training. Four themes emerged: experiences of medical students with generative AI, perceived benefits of AI, concerns about its use, and recommendations for responsible AI use. The study highlights AI use among medical and nursing students from LMIC setting. However, data collection was conducted from November 2023 to January 2024. As a result, our findings may be limited given the rapidly changing information on AI use.

Students had diverse experiences with using AI. They used it not only for medical education but also for learning other skills, entertainment, and emotional support, among others. This represents the capability of generative AI to do a wide variety of tasks. An analysis of ChatGPT usage from 2022 to 2025 revealed that it is used for a variety of tasks, like giving information, creating media content, and offering technical support, among others [19].

In their medical education, students used AI sources for completing their assignments, online examinations, and writing their research proposals. This was similar to quantitative findings in Uganda, where students used AI sources for completing assignments (56%) and examinations (35%) [15]. This suggests a potential misuse of AI sources and the likely examination malpractice from AI use [11,20].

There were several benefits of AI. AI sources provided easy, tailor-made, and simplified information for students compared to traditional textbooks. Therefore, blending the inherent advantages of AI sources with the traditional sources of information may facilitate students’ comprehension, learning, and long-term retention of medical knowledge. In another study in Saudi Arabia, faculty and students reported that AI had a positive contribution to education [20].

Students found it easy to confide in AI sources for their academic and emotional needs. While students felt that they could not approach their faculty or fellow students with questions, AI sources were seen to provide human-like and appropriate responses in line with their emotional needs. Similar studies have equally reported students’ use of AI for their mental health needs [15]. AI sources have been suggested for use among youths with mental health challenges in settings with a shortage of healthcare professionals [21], which suggests a role that AI may play in mental health. In Sweden, young people with emotional needs perceived AI as readily available and accessible to them [22].

Beyond the benefits, the students shared experiences that demonstrated the limitations of AI. They noted that the information from AI sources was reliable and accurate; however, it’s occasionally incorrect. Some of the students who used AI for completing assignments or writing research proposals noted that they did not pass their courses because of AI use. Previous studies have noted concerns of accuracy among AI sources, with some noting how they may make up information [20,23–25]. Concerns of accuracy made some students and faculty perceive AI as not a credible and reliable source of information for medical students [26].

Besides accuracy, AI sources were perceived to provide basic information, which was devoid of detailed, systematic, and comprehensive information. Consequently, students who primarily relied on AI sources were perceived as unqualified to attend to patients. Students also feared that using AI for completing assignments and examinations may lead to laziness, examination malpractice, and loss of critical thinking, scholarly writing skills, and creativity [26]. These are threats recognized by many other scholars. There is no evidence on whether using AI reduces clinical competency; however, several studies report a decline in cognitive capabilities with increased AI use [27–29].

In our study, students expressed fears about job loss. This is similar to concerns of AI replacing human interaction and its likely effect in reducing the number of healthcare providers [23]. Recommendations from the students included cautious use of AI, complementing it with other sources of information, and learning how to use it. They advised changes in assignment formats and the use of AI to curb the risk of AI misuse and examination malpractice. However, some AI sources may be difficult to detect even with the use of AI and plagiarism detectors. Students are often more knowledgeable about AI use than the faculty, which underscores how they may circumvent processes to detect AI misuse [26]. This highlights the likely difficulty in regulating the integrity of online examinations and assignments following the advent of AI.

## Conclusion

In our study, health professions students used AI sources as the initial source of information, alongside other resources, and as a last resort for their learning. Students widely used AI to enhance their learning, prepare for tutorials, write their research proposals, and complete assignments and online examinations. Students perceived AI sources to be more accessible, efficient, and relevant for their academic and non-academic needs. Students expressed concerns related to the accuracy of information, its negative effect on critical thinking and creativity, and fears of examination malpractice. Our study findings maybe valuable in formulating policies on responsible use of AI in medical education. The inevitable use of AI in medical and nursing educations calls for integration of AI in the medical curriculum and training to harness its benefits in medical and nursing education.
